# Design, Fabrication, and Characterization of a Planar Three-Electrode Trigger Switch Based on Flexible Printed Circuit Process

**DOI:** 10.3390/mi15050586

**Published:** 2024-04-28

**Authors:** Pengfei Xue, Peng Xiong, Heng Hu, Tao Wang, Mingyu Li, Qingxuan Zeng

**Affiliations:** 1 State Key Laboratory of Explosion Science and Technology, School of Mechatronical Engineering, Beijing Institute of Technology, Beijing 100081, China; xuepengfei95@126.com (P.X.);; 2 China Ship Development and Design Center, Wuhan 430064, China

**Keywords:** flexible printed circuit, three-electrode planar trigger switch, exploding foil initiator system

## Abstract

An exploding foil initiator system (EFIs) is essential in modern weaponry for its safety and reliability. As the main component of EFIs, the performance of the switch is critical to EFIs. In this study, a planar three-electrode trigger switch was designed and fabricated using the Flexible Printed Circuits (FPC) process. Subsequently, the performance of the FPC switch was tested. The results show that the self-breakdown voltage of the FPC switch is stable. In addition, an FPF switch with a 0.6 mm main electrode gap demonstrated consistency, with delay times below 31.75 ns, and a jitter ranging from 1.7 ns to 10.94 ns at 900 V to 1200 V, evidencing the FPC switches’ reliability and uniform performance across various voltages. Compared to the Micro-Electro-Mechanical Systems (MEMS) switches of similar dimensions, the FPC switches achieved a faster high-current attainment with less inductance, showing a 5% reduction in loop inductance. The repetitive testing results demonstrate that the FPC switch maintains consistent output performance, with stable peak currents, peak current time, and delay time over 50 action cycles, highlighting its repeatability. The FPC switch was assembled with an EFI chip and capacitor into an integrated system, which was subsequently able to successfully detonate HNS-IV at 1000 V/0.22 μF, proving the FPC switch’s potential in low inductance applications.

## 1. Introduction

An exploding foil initiator system (EFIs) is an inline detonation or ignition device. J.R. Stroud [1] first proposed the concept of incorporating a layer of flyer and a hollow cylindrical shear into an energy-containing bridge membrane, leading to the creation of the earliest inline initiator system, commonly referred to as the slapper detonator. This system offers an advantage over traditional ignition systems in that the EFIs does not directly contact the explosives. Instead, it can only be initiated under specific high-impulse current conditions. This feature greatly enhances safety and reliability [2,3], thus the EFIs has found widespread application in weapons ignition and detonation systems.

Understanding the working principle of the EFIs [4], it is evident that the discharge circuit unit needs to rapidly release the electrical energy stored in the capacitors to the load within an extremely short period (around a few hundred nanoseconds). Thus, the high-voltage switch becomes crucial in the entire discharge unit. Various types of high-voltage switches are employed for EFIs, including a spark gap switch, a metal oxide semiconductor-controlled thyristor (MCT) switch, and a planar switch [5]. The spark gap switch, which includes a gas switch and vacuum switch, are less affected by high temperatures, high voltages, and strong radiation due to their specific atmosphere or vacuum environment. However, their larger size challenges the miniaturization of the EFIs [6,7].

An MCT switch [8] consists of a semiconductor gate current tube and a pair of complementary metal oxide semiconductor field-effect transistors (MOSFETs)—specifically, P-MOSFETs and N-MOSFETs, which are used for turning on and off, respectively. Compared to the spark gap switch, the MCT switch offers greater stability and reliability, smaller size, and reusability. They have found applications in electronic security systems and low-energy EFIs and have been implemented in model weapon systems [9,10,11,12]. However, the trigger threshold of an MCT switch is low and easily affected by interference signals and ambient temperature.

In order to meet the demands of a miniaturized EFIs, researchers have developed a planar switch based on the operating principle and structure of a vertical spark gap switch [13,14,15]. The planar switch also consists of three electrodes: the positive, negative, and trigger. Compared with the vertical spark gap switch, the planar switch has greatly reduced the switch volume and can be directly integrated into the pulsed power unit using a micromachining process [16]. The development of the planar switch has led to a breakthrough in achieving miniaturization in a EFIs.

In recent years, the emergence and application of new processing technologies, such as the Low-Temperature-Co-fired-Ceramic (LTCC) process and Printed Circuit Board (PCB) technology, have led to a new development direction for the integration of EFIs [17,18,19,20]. Researchers prepared the planar three-electrode high-voltage switch using the LTCC process and PCB technology. In addition, they integrated the switch with the EFI chip to obtain the EFIs integrated device. The planar switch prepared using these new technologies can better realize the integration with the EFIs. In addition, the development of switch preparation techniques as well as materials in different fields provides more potential options for switches in EFIs [21,22,23,24].

In general, the planar trigger switches are distinguished by their compact profiles and their seamless amalgamation into integrated EFIs, attributes that establish them as the preferred switch type in such application. During the conduction process, the planar trigger switch inevitably experiences electrode ablation, which poses a concern for the switch’s performance upon subsequent reuses. However, the inherent design of EFIs as disposables has led to a dearth of research on the reuse capabilities of planar switches. To enhance the reliability and detectability of the system, it is crucial to examine both the continuity of the circuit and the magnitude of electrical discharge to ensure it meets the design requirements of the metal bridge foil electrical explosion. This necessitates the system circuit to support repeatable charging and discharging, requiring the planar switch to be reusable while maintaining consistent output performance.

Therefore, in this study, the planar three-electrode trigger switches were fabricated using FPC technology. Their morphology dimensions and related properties were characterized to ensure the switches’ electrical properties align with the stringent requirements of planar switches for the exploding foil initiation system. The service life of the FPC switch was evaluated through repeated conduction experiments. Subsequently, the integration of the FPC switch with the exploding foil chip and capacitor was carried out, followed by detonation experiments to confirm the practicality of the FPC switch. The planar switch designed in this paper has the following advantages: The FPC process allows for the mass production of planar switches, which greatly reduces the manufacturing cost of switches. FPC switches are less than 100 μm thick and have a negligible volume, which allows for a more flexible integration with the EFIs wiring. This flexibility shortens the length of the discharge circuit and subsequently reduces the circuit inductance, resulting in improved performance. In addition, the FPC switches are repeatable and can be guaranteed to have a stable output performance during operation. The advantages of the FPC switch contribute to achieving the miniaturization of the EFIs and a low-energy development.

## 2. Design and Fabrication of the Planar Three-Electrode Trigger Switch

**Figure 1:**
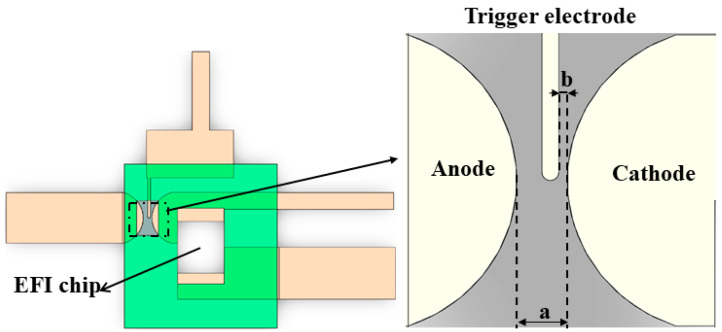
The diagram of the planar trigger switch.

The structure of the planar switch in this study is shown in Figure 1, which consists of the positive electrode, negative electrode, and trigger electrode. The top surface of switch is covered with a polyimide film, while the bottom surface is covered with a metal layer. In addition, welding sites are reserved for integration with the EFI chip as Figure 1 shows. The parameter ‘a’ in Figure 1 represents the distance between the gap of the main electrodes, and ‘b’ represents the distance between the trigger electrode and the negative electrode. According to Bashin’s law [25], when the air pressure inside the planar switch and the inter-switch gap are determined, the switch’s self-breakdown voltage becomes a fixed value. Furthermore, the operating voltage of the switch was generally limited to 60% to 90% of the self-breakdown voltage. Based on previous research conducted in this laboratory [15], the main gap distance “a” was designed to be 0.6~1.0 mm, and the distance between the negative electrode and the trigger electrode “b” was 100 μm, which can meet the trigger voltage 1000~2000 V operating requirements.

The switch production via FPC technology follows a systematic methodology:

(1) The FPC substrate undergoes mechanical modification, where it is shaped by punching out general forms and creating holes.
(2) These perforations are then metalized, typically through electroplating, to ensure bidirectional conductivity across the FPC board.
(3) Employing techniques akin to those used in MEMS manufacturing, the switch design is transferred onto the FPC substrate using a sequence of lithographic exposure, development, etching, and removal of the photoresist.
(4) Strategic application of a protective film comes next, defining areas to be shielded during subsequent processing.
(5) The final stage involves the lamination of the reinforcement material, cover film, switch layer, and adhesive, which are bonded under conditions of elevated temperature and pressure to form a unit.

**Figure 2:**
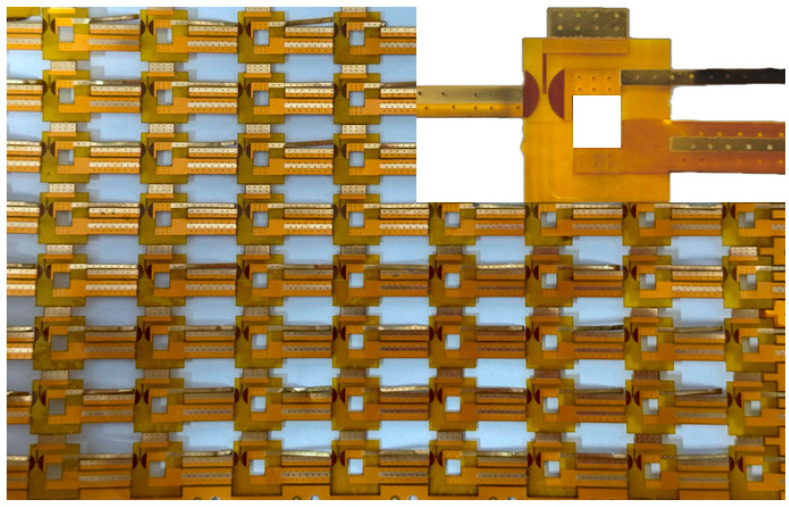
Physical drawing of the planar trigger switch based on FPC.

Figure 2 illustrates the planar trigger switch crafted using the FPC method. This process enables the mass production of the switch at reduced manufacturing costs.

**Figure 3:**
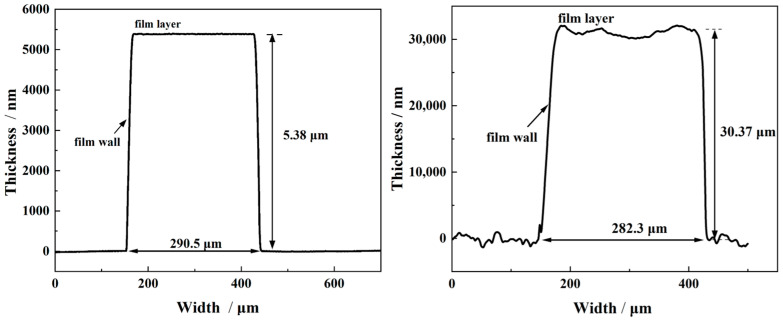
Surface profile of MEMS switch (5.38 μm) and FPC switch (30.37 μm).

To evaluate the dimensional characteristics of the prepared planar switch, the dimension of both the FPC switch and MEMS switch were measured using a surface profiler (Dektak 150, Veeco, New York, NY, USA). The planar switches with a main electrode gap distance of 0.6 mm were examined. The test results, as shown in Figure 3, demonstrates that the main electrode gap distance of the planar switch prepared based on the FPC process is 643 μm with an error of 7.2%. The film thickness is 30.37 μm, and the trigger electrode width is 282.3 μm with an error of 5.9%. The gap distance of the main electrode of the MEMS switch is 627.2 μm, the film thickness is 5.38 μm, and the width of the trigger is 290.5 μm. Additionally, the surface roughness of the switch was measured. The test results indicate that the roughness of the FPC switch is 366.1 nm, while the roughness of the MEMS switch measured 5.82 nm. The reason for this discrepancy is that the metal layer of the FPC switch was prepared using electroplating, which offered faster film formation rates and produced a thicker metal layer in comparison to the magnetron sputtering coating method employed for the MEMS switch.

## 3. FPC Switch Characterization and Performance Test

**Figure 4:**
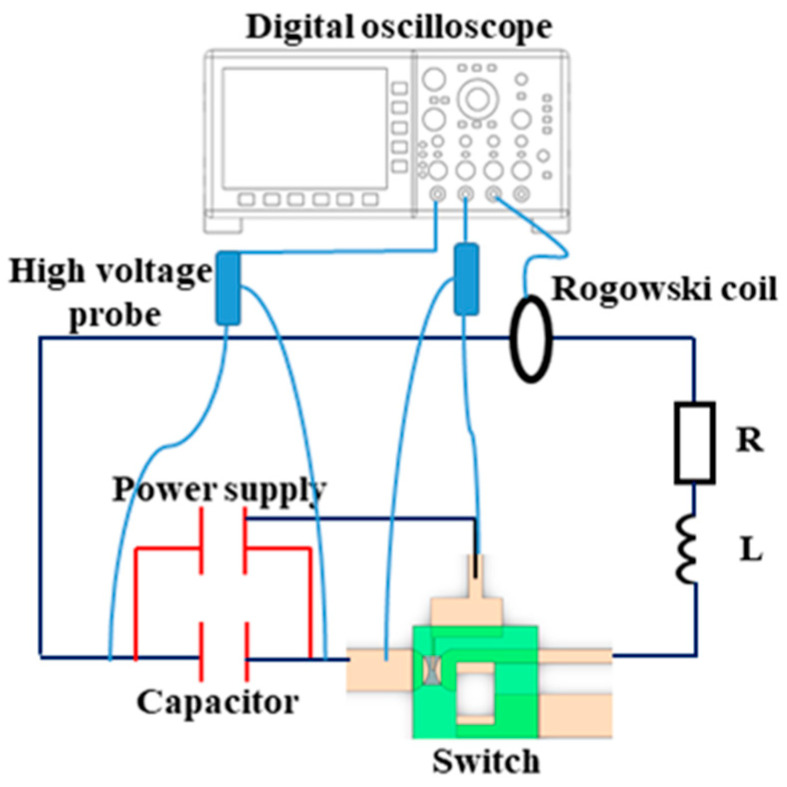
Experimental device for performance testing of planar trigger switch.

In order to investigate the performance of the planar trigger switch, the test rig was set up as shown in Figure 4, which consists of a switch test circuit, a Roche coil current loop (5046, Pearson, San Francisco, CA, USA), a high-voltage probe (P6139B, Tektronix, Beaverton, OR, USA), and a digital oscilloscope (DPO3034, Tektronix, Beaverton, OR, USA) used to record the voltage and current signals. The test circuit includes a high-voltage power supply (Sibake, Beijing, China), a capacitor (Hong Ming Electronics, Chengdu, China), and an equivalent resistance. The high-voltage power supply was connected to the positive and negative terminals of the capacitor to supply power, while the trigger terminal was connected to the FPC switch. A Roche coil was connected to the circuit to test the current flowing through it. The probe was connected to the two ends of the capacitor and planar switch to test the voltage signals.

The operational performance of the planar trigger switch is intricately influenced by environmental conditions such as ambient temperature, humidity, atmospheric composition, and barometric pressure. In this study, experiments were meticulously carried out under controlled conditions: a temperature of 25 °C, relative humidity of 50%, an air-filled environment and standard atmospheric pressure.

### 3.1. Self-Breakdown Voltage Test

The self-breakdown voltage of the planar switch depends on factors such as the switch electrodes, the type of atmosphere, and the gas pressure. In this work, the focus of the test was on the effect of the main electrode gap on the self-breakdown voltage of the planar switch.

To investigate the effect of the main electrode gap on the self-breakdown voltage, the main electrodes of the planar switch were connected to the positive and negative poles of a capacitor. Subsequently, the high-voltage power supply gradually charged the capacitor at a rate of 500 V/min. During this voltage rise, if a breakdown phenomenon occurred, it was considered a self-breakdown, and the voltage detected using the high-voltage probes at both ends of the planar switch at that moment was recorded as the self-breakdown voltage. In this paper, the self-breakdown voltage was tested for the FPC switch with the main electrode gaps ranging from 0.6 mm to 1.0 mm. Each type of switch was tested five times and the minimum value of the breakdown voltages was taken as the self-breakdown voltage.

### 3.2. The Delay Time of the Switch Test

The working principle of the planar trigger switch is that the main electrodes are initially charged to a certain voltage, which is lower than the break voltage between the main electrodes. When an impulse voltage is applied to the trigger electrode, it creates a shorter gap between the trigger electrode and the adjacent main electrode. This leads to the initiation of charged particles, which then accelerate under the influence of the electric field between the main electrodes. Eventually, the main electrodes break down, and the switch starts conducting.

**Figure 5:**
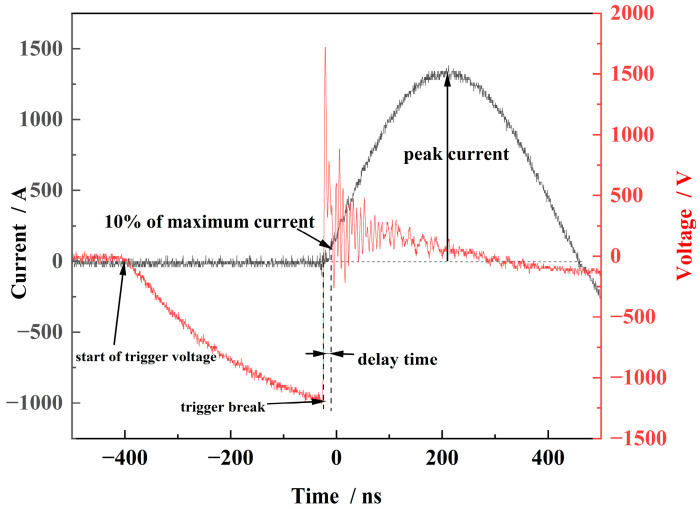
Typical signal for switch delay time (a = 0.6 mm).

During the testing process, a high-voltage probe was connected to the trigger terminal and the negative terminal of the switch. The current profile in the circuit was measured using a Roche coil current loop. Figure 5 illustrates a typical signal diagram of the switch delay time. In this study, the delay time of a planar switch with a main electrode gap of 0.6 mm was tested at different voltages. The delay time of the switch was measured as 10% of the time from the start of the trigger voltage fallback, as shown in Figure 5, to the time when the current in the circuit reached its peak current.

### 3.3. The Intrinsic Inductance and Resistance Test of the Switches

The inductance and resistance in the circuit of the EFIs play a crucial role in determining the energy utilization rate of the system. A lower inductance and smaller resistance in the circuit results in a higher amount of energy being directed towards the electric explosion process of the bridge foil. The inductance and resistance in EFIs come from the wires in the loop, the planar switch, capacitor, EFI chip and solder joints, making them important factors for evaluating the quality of the switch.

In the discharge circuit, the inductance and resistance can be simplified and modeled as an RLC discharge circuit. The circuit equation governing the behavior of the RLC discharge circuit was as follows [26]:

(1) $$\frac{1}{C}\int idt+L\frac{di}{dt}+Ri=U_{0}$$

where: *C* was the capacitance capacity, µF; *U*_0_ is the operating voltage, V; *R* is the loop resistance, mΩ; *L* is the loop inductance, nH; *i* was the circuit current, A.

Solving the above equation gives:

(2) $$i\left(t\right)=\frac{U_{0}}{\omega L}e^{-\delta t}\sin\omega t$$

(3) $$\omega =\sqrt{\frac{1}{LC}-\frac{R^{2}}{4L^{2}}}$$

(4) $$\delta =\frac{R}{2L}$$

The first peak current *I*_1*max*_, the second peak current *I*_2*max*_, and the corresponding first period of current *T*_1_ were obtained from the current curve, and solving the above equations results in:

The inductance and resistance in the circuit can be calculated from Equation (5).

(5) $$\left\{ \begin{array}{l} L=\frac{T_{1}^{2}}{C}{\left[4\pi^{2}+\left(\ln\frac{I_{1\max}}{I_{2\max}}\right)\right]}^{-1}\\ R=\frac{2L}{T_{1}}\ln\frac{I_{1\max}}{I_{2\max}} \end{array}\right.$$

### 3.4. Reusable Test of FPC Switch

The efficacy of the EFIs, which is designed for single-use operation, largely depends on its circuit’s ability to efficiently charge and discharge; this capability is crucial for ensuring system reliability. Traditionally, the functionality of the EFIs was assessed merely by checking the circuit’s on/off status, an approach that significantly undermines its reliability due to inadequate diagnostics. To enhance safety and reliability, it is imperative to augment the system’s diagnostic capabilities. This can be achieved by equipping the circuit within the EFIs with the ability to conduct repeated charging and discharging cycles. This requirement necessitates a circuit design that can safely bypass the EFI chip, ensuring the switch incorporates a repeatable and stable functionality. 

In this work, to evaluate the service life of FPC switches, switches with the main electrode gaps of 0.6 mm and 1.0 mm underwent testing through repeated actions until failure. Throughout the test, key electrical parameters were recorded after each switch activation, including *t_I_*_max_ (peak current time), *I*_max_ (peak current), and *t*_delay_ (the switch delay time).

### 3.5. Firing Test of FPC switch

**Figure 6:**
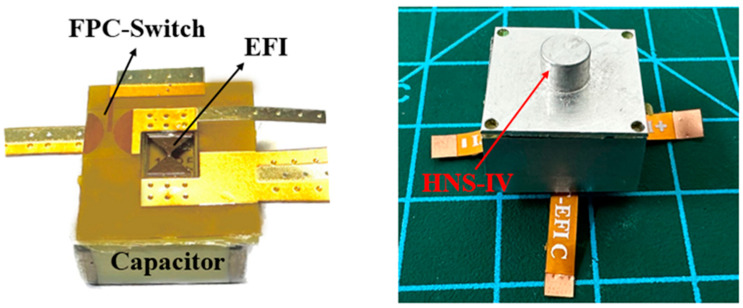
FPC switch integrated exploding foil initiation system.

The key application for the planar trigger switch is for the EFIs which function to initiate the charge. Thus, to assess its practical utility, the FPC switch (a = 0.6 mm) was integrated with a capacitor (0.22 μF) and an EFI chip (prepared using MEMS technology) for detonation testing. Figure 6 illustrates the integration of the EFIs with an FPC switch. The detonation capability is tested using HNS-IV (Xi’an Qinghua company, Xi’an, China) with a density of 1.57 g/cm^3^, validating the switch’s explosive performance in the setup.

## 4. Results and Discussion

### 4.1. Self-Breakdown Voltage of the FPC Switch

**Figure 7:**
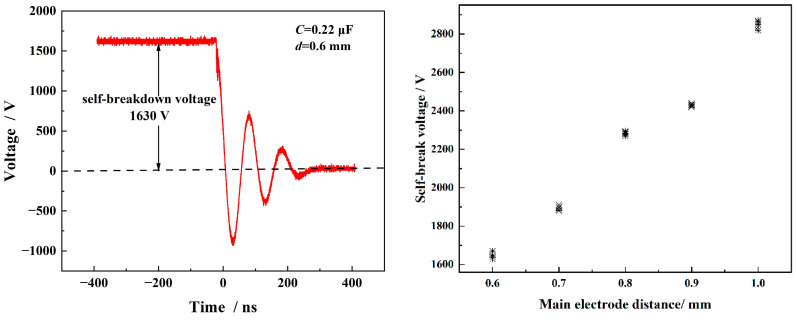
Typical self-breakdown voltage signal and self-breakdown of switches with different main electrode gaps.

Figure 7 shows a typical plot of the self-breakdown voltage signal and voltage distribution for switches with different main electrode gaps. The self-breakdown voltage data for different main electrode gap distances are presented in Table 1. The data in Table 1 demonstrate that, under constant conditions, the self-breakdown voltage of the planar trigger switch increased with the increase in the main electrode gap distance. The self-breakdown voltage was recorded as 1630 V when the main electrode gap was 0.6 mm. In addition, the self-breakdown voltage jitter of the switches with different main electrode gap distances are all less than 3%, signifying that the batch-manufactured FPC switches exhibit excellent uniformity and consistently stable output performance.

### 4.2. The Delay Time of the FPC Switch

**Figure 8:**
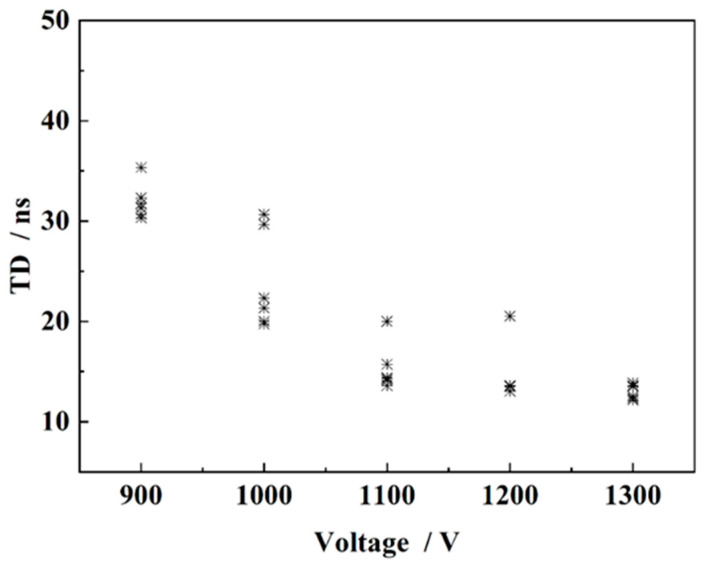
Switch delay time under different operating voltage (a = 0.6 mm).

The delay time and the jitter of the FPC switches can be observed from Figure 8, when the main electrode gap was kept constant and the switch delay time decreased as the operating voltage increasing. The average delay time experienced a faster change within the range of 900 V to 1100 V, decreasing from 31.95 ns to 15.37 ns. Additionally, as the operating voltage approached the self-breakdown voltage, the change in the average delay time became smaller. Furthermore, the switch jitter time of the FPC switch at different voltages was more stable and was in the range of 1.7 ns to 10.94 ns, indicating that the switch had high homogeneity and reliability at different voltages.

### 4.3. The Intrinsic Inductance and Resistance of the FPC Switch and MEMS Switch

**Figure 9:**
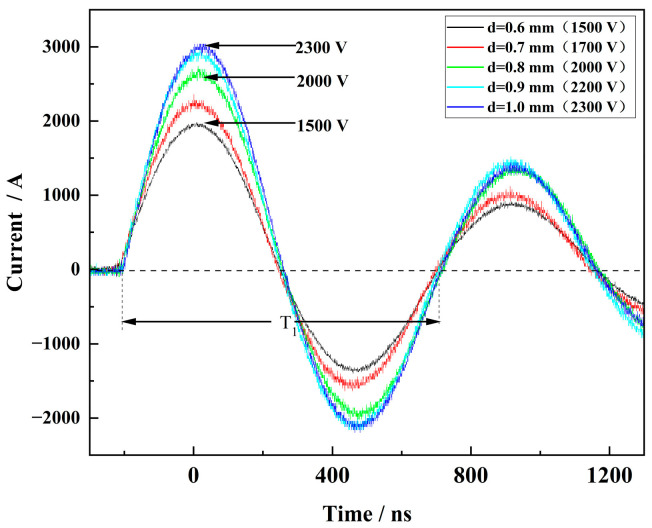
Short-circuit loop current curves for FPC switch.

The inductance and resistance in the circuit can be calculated from Equation (5). In this paper, the short circuit current data of the FPC switch are presented in Figure 9. Additionally, the circuit data of the FPC switch and the same size of MEMS switch are summarized in Table 2. From the results, it is observed that the inductance of the FPC switch in the same circuit is reduced by 5% compared to that of the MEMS switch. Moreover, the first peak currents in the FPC switch circuits are consistently higher than those in the MEMS switch circuits under the operating voltage range of 1500 V to 2300 V. For instance, at an operating voltage of 1500 V, the FPC-based circuit reaches a peak current value of 1960 A after 219.6 ns, while the MEMS-based circuit reaches a peak current value of 1790 A after 226.0 ns. This indicates that under identical operating voltage and main gap conditions, the FPC switch circuit is capable of achieving a higher peak current in a shorter time, which facilitates the release of energy stored in the capacitance and increases the current density in the metal bridge area during the electrical explosion process [19]. These findings highlight the advantages of the FPC switch in terms of faster energy release and higher current density, making them more suitable for applications requiring an efficient utilization of energy.

In conclusion, the FPC-planar switch offered significant advantages for meeting the requirements of the EFIs. Compared to the MEMS switch, the FPC switch maintained a similar switch performance while enabling a shorter connection circuit in the EFIs. This led to a reduction in both inductance and resistance in the circuit, promoting the development of low-energy and miniaturized EFIs.

### 4.4. Reusable Characteristics of FPC Switch

**Figure 10:**
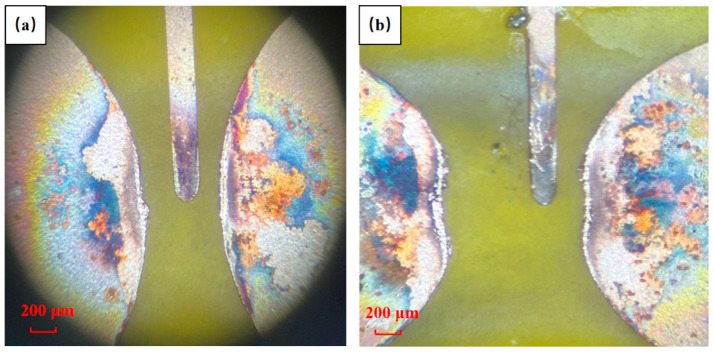
Electrode ablation after reuse of switches: (**a**) FPC switch with 0.6 mm main electrode gap; (**b**) FPC switch with 1.0 mm main electrode gap.

**Figure 11:**
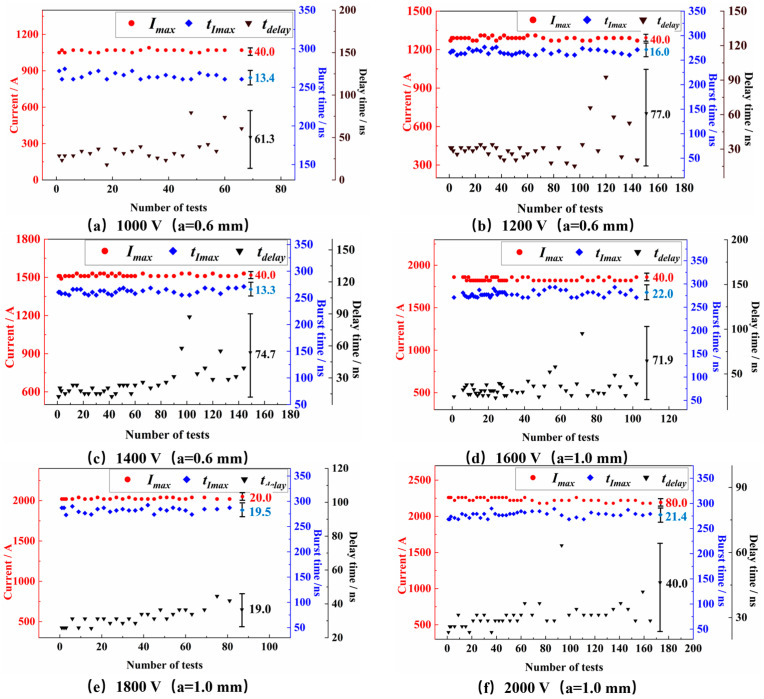
FPC switch reusable electrical characteristics at different voltage conditions.

The ablation of the switch due to repetitive use is shown in Figure 10. Electrode endurance in planar switches is contingent upon the metal layer’s thickness. Enhanced thickness acts as a bulwark, diminishing the propensity for deformation in both the main and trigger electrodes from spark-driven ablation during conduction. Figure 11 illustrates the electrical characteristic signal plot of the FPC switch after repeated use. Additionally, Table 3 presents the electrical characteristics of switches with 0.6 mm and 1.0 mm main electrode gaps at voltages ranging from 1000 V to 1400 V and 1600 V to 2000 V, respectively. The results include the number of switch onsets (the number of times the switch fails for the first time at that voltage), peak current, time to peak current, delay time, and the corresponding jitter value (polar difference).

The number of FPC switch actions does not follow a consistent trend with increasing operating voltage, as observed in Table 3. For 0.6 mm switches, the counts rose from 70 to 147 and then decreased to 144 as the voltage escalated from 1000 V to 1400 V. Similarly, the number of switching actions of 1.0 mm switches drops from 103 at 1600 V to 83 at 1800 V, then surges to 170 at 2000 V. This irregular pattern can be attributed to the principle of the planar switch [16]. As can be seen from Figure 10, electrode ablation occurs during repetitive cycles, intensifying with higher operating voltages. This phenomenon widens the gap between the main electrodes, hindering conductivity channel formation. On the contrary, escalating voltage boosts the electric field between the electrodes, facilitating discharge channel creation. Thus, the service life of the FPC switch is determined via the coupling effect of the ablation condition of the switch electrodes and the operating voltage.

In addition, Figure 11 shows that the loop peak current and peak current time after repeated triggering do not produce large fluctuations, indicating that the FPC switches have a good anti-burnout performance within a certain number of times of use, and they can maintain a more stable output performance. With the increase in the number of actions, the delay time of the FPC switch under different action conditions shows a similar pattern: The switch is more stable in terms of delay time and exhibits a slight jitter within 50 actions. When the switch action is more than 50 times, the switch on-time delay time shows a larger jitter and the switching delay time starts to increase. This is because the switch electrodes are deformed by the spark ablation after 50 times, and the electric field uniformity is affected by corona discharge and the polarity effect which leads to the delayed on-state of the switch [27]. Overall, the FPC switches meet the requirements for reuse over a wide range of voltages.

### 4.5. Applications of FPC Switch

Experimental results show that the FPC switch integrated EFIs can successfully detonate the HNS-IV, under the minimum firing requisites of 1000 V/0.22 μF. The firing energy is significantly lower compared to circuits with planar switches based on MEMS, LTTC and PCB technologies [15,19,20]. The success of the detonation has proved the usefulness of FPC switches; in addition, the use of FPC switches in the EFIs also has the following advantages: Firstly, the adoption of the FPC switch inherently abbreviates the circuit path, consequently diminishing the circuit’s inductive properties and attenuating the attendant energy losses. Secondly, the EFIs fortifies its chip units with MEMS technology. The deposition of the metallic stratagem was executed via magnetron sputtering, ensuring a dense metal film with low roughness and uniform thickness. Therefore, the metal bridge foil has a better consistency when an electric explosion occurs, and can reduce the energy loss caused by the poor densification and roughness of the metal film. By combining the respective advantages of the two technologies, the FPC-based explosion foil integrated initiator device has a lower ignition voltage and ignition performance is more stable.

## 5. Conclusions

The study findings indicate that the FPC switch offers advantages such as low inductance, reusability, and facilitating integration with the EFIs. Specifically, the intrinsic inductance of FPC switches decreased by 5% when compared to MEMS switches. FPC switches with varying main electrode gaps reliably function up to 50 times across their voltage range without significant fluctuations in key circuit parameters like peak current, peak current time, and delay time. Even after over 50 cycles, peak current and peak current time remain consistent, with only a slight increase in delay time jitter. The integration of FPC switches with the EFIs has significantly shortened the circuit, enabling successful detonation of HNS-IV under the condition of 1000 V/0.22 μF. The intrinsic resistance of FPC switches is not significantly reduced compared to MEMS switch, however, the circuit resistance remains significant, pinpointing an area for subsequent research. In addition, the application of FPC switches for the charge/discharge detection of the EFIs will be investigated to achieve non-destructive detection before the action of the EFIs.

## Figures and Tables

**Table 1:** Self-breakdown voltage for FPC switches with different main electrode gap.

Switch gap distance/mm	0.6	0.7	0.8	0.9	1.0
Self-breakdown voltage/V	1630	1880	2270	2420	2820
Jitter/V	40	30	25	20	50

**Table 2:** Calculated resistance and inductance of discharge circuit for FPC switch and MEMS switch.

Switch	Gap Distance/mm	Operating Voltage/V	*I*_1max_/A	*I*_2max_/A	T_1_/ns	L/nH	R/mΩ
FPC switch	0.6	1500	1960	900	910.1	93.9	160.6
	0.7	1700	2280	1040	909.0	93.8	161.8
	0.8	2000	2700	1340	912.1	94.1	145.0
	0.9	2200	3000	1480	909.0	94.0	146.1
	1.0	2300	3040	1400	899.0	93.7	159.9
MEMS switch	0.6	1500	1790	1000	927	98.1	123.2
	0.7	1700	2100	1120	922	96.9	132.1
	0.8	2000	2420	1220	927	97.8	144.5
	0.9	2200	2620	1240	933	98.9	158.5
	1.0	2300	2740	1280	938	99.0	161.3

**Table 3:** Electrical characteristics of FPC switch for reuse.

Gap Distance/mm	Operating Voltage/V	Conduction Number	Average of *I*_max_/A	Jitter of *I*_max_/A	Average of *t_I_*_max_/ns	Jitter of *t_I_*_max_/ns	Average of *t*_delay_/ns	Jitter of *t*_delay_/ns
0.6	1000	70	1064.8	40	264.9	13.4	36.3	61.3
	1200	147	1288.3	40	266.7	16	31.6	77.0
	1400	144	1515.6	40	261.9	13.3	25.9	74.7
1.0	1600	103	1832.8	40	278.9	22	34.3	71.9
	1800	83	2026.9	20	282.6	19.5	32.0	19.0
	2000	170	2230.0	80	277.5	21.4	30.8	40

## Data Availability

The original contributions presented in the study are included in the article, further inquiries can be directed to the corresponding author.
